# Development and validation of The Breaking Bad News Attitudes Scale

**DOI:** 10.1186/s12909-021-02636-5

**Published:** 2021-04-07

**Authors:** Kátia Laureano dos Santos, Paola Gremigni, Giulia Casu, Victor Zaia, Erik Montagna

**Affiliations:** 1 Centro Universitário FMABC, Faculdade de Medicina do ABC, Av Príncipe de Gales, 821, Santo André, 09060-650 Brazil; 2 Department of Psychology, University of Bologna, viale Berti Pichat, 5, 40127 Bologna, Italy; 3 Centro Universitário FMABC, Instituto Ideia Fértl de Saúde Sexual e Reprodutiva, Av. Príncipe de Gales, 821, Santo André, 09060-650 São Paulo Brazil

**Keywords:** Breaking bad news, Medical education, Assessment, Validation, Pediatricians, SPIKES

## Abstract

**Background:**

Communication of bad news plays a critical role in the physician-patient relationship, and a variety of consensus guidelines have been developed to this purpose, including the SPIKES protocol. However, little is known about physicians’ attitudes towards breaking bad news and to be trained to deliver it. This study aimed to develop and validate a self-report questionnaire to assess physicians’ attitudes towards principles of the SPIKES protocol and training on them.

**Methods:**

The Breaking Bad News Attitudes Scale (BBNAS) was administered to 484 pediatricians and 79 medical students, recruited at two scientific conferences and two medical schools in Brazil. The questionnaire structural validity, reliability, and associations with other variables were tested.

**Results:**

The BBNAS showed adequate validity and good reliability, with two factors measuring attitudes towards the SPIKES strategy for braking bad news (α = 0.81) and the possibility to be trained on it (α = 0.77), respectively.

**Conclusion:**

The novel questionnaire is a psychometrically sound measure that provides information on physicians’ agreement with the SPIKES protocol. The BBNAS can provide useful information for planning training and continuing education programs for clinicians on communication of bad news using the SPIKES as a framework.

**Supplementary Information:**

The online version contains supplementary material available at 10.1186/s12909-021-02636-5.

## Background

In the context of medical communication, bad news has been seen as any information that negatively and seriously alters the patient’s view of his or her future, even if temporarily [1]. Bad news not only refers to death [2] but also to diagnoses that impose changes in the patient’ life [3]. Breaking bad news (BBN) is an important part of every physician’s clinical practice, but it might be a burden for both the patient and the physician, with difficulties in this communication negatively affecting both of them. Performed improperly, BBN can lead to patient’s stress, anxiety, and misunderstanding of diagnosis, treatment, and prognosis, resulting in less favorable outcomes overall [4, 5]. Further, physicians’ psychophysiological stress reaction in medical communication of bad news can lead to an increase in their anxiety, burnout, and alienation from the situation and the patient [6]. However, grief also can result in burnout and decreased mental health, what may lead to an erroneous interpretation of grief as stress, anxiety or depression [7].

BBN is even more critical in pediatrics, as the patient is not the recipient of the communication nor is able to understand the information given and its consequences [8]. Besides, misunderstandings might derive from the fact that parents never expect a child not to be healthy [9]. Empathic and effective communication with the family is, therefore, essential in pediatrics since the family has an influence on the clinicians’ actions and their relationship with the patient [10, 11]. Moreover, pediatricians are highly involved with the patient’s family, and the care for children in life-threatening circumstances is more likely to cause impairments in the mental health condition of the physician and health care professionals [7].

In recent years, a variety of consensus guidelines for communicating bad news to patients have been published [11–15]. One of the most used protocol for delivering bad news is the SPIKES, [16] a six-step strategy that facilitates the information flow and addresses the patient’s distress. Although the SPIKES protocol has been developed by oncologists to delivering bad news to cancer patients [17], it has been considered appropriate and used in other health care areas as well [18, 19].

Several studies have shown the beneficial effects of implementing a guidance on how to systematize breaking bad news in enhancing physicians’ self-confidence and abilities and reducing anxiety concerning BBN [20–22]. Protocols and guidelines such as the SPIKES seem to be crucial tools for the proper delivering of bad news [20], and the specific choice for the SPIKES as model is due to the fact that it is the most widely cited [23], and the one that provide a framework that improve skill acquisition for bad news communication (BNC) [24]. However, their implementation is a work in progress in curricula of medical courses and continuing medical education [25, 26], and many gaps have been addressed in how clinicians are prepared to have difficult conversations [27–30]. Indeed, although the SPIKES protocol has been rated as highly acceptable by patients, there is a mismatch between the doctors’ and patients’ views with how clinicians facilitated the diagnostic discussion, and also the patients’ perception is much varied [31].

Although the controversy over whether it is possible to develop BBN abilities [32], several studies have reported that training medical students and clinicians can have positive effects on their interest in acquiring the desired skills [2, 23, 33]. Attitudes towards properly communicating bad news and the possibility to be trained on desirable skills by medical students and physicians are preliminary steps to develop appropriate medical courses that can effectively change their behavior [34]. However, physicians’ attitudes towards breaking bad news and learning of the related skills has not been thoroughly investigated.

Despite the fact that other tools were developed in order to assess medical communication [35, 36], content knowledge regarding protocols for BNC [37, 38], and other protocols for BNC [13, 14], the tool herein proposed is oriented to assess the physician’s attitudes regarding the principles of the SPIKES protocol and the promptness to training the BNC skills, more than communication skills in general. In this study attitude differs from the observed behavior. Here we consider attitude as the interrelation between beliefs, feelings and behaviors, also considering the conative dimension of attitude, that is, the tendency to act in a determined way [39]. In this way, the instrument proposed aims to verify this behavioral tendency, regarding the assent or not to possible behaviors.

Finally, the choice for pediatricians as the main professional was because the characteristics and particularities of BNC for this audience is difficult in many ways, because it is a “person-oriented” specialty [40] and are reported to have deeper involvement with patients and families [41]. Thus, in this study were involved especially pediatricians besides medical students, despite the tool is not proposed exclusively for pediatrics. The present study aimed to develop and validate a psychometric scale for evaluating clinicians’ attitudes towards delivering bad news according to the principles underlying the SPIKES protocol and the training to learn the desired skills.

## Methods

### Participants and data collection

The sample size was defined through a minimum of 10 participants per item and 100 per factor for exploratory factor analysis (EFA) and a sufficient number of cases to run confirmatory factor analysis (CFA) and for the CFA model to converge without improper solutions [42].

Data collection was carried out between November 2018 and June 2019, mainly at two national pediatric conferences and two medical schools in southeast and northeast of Brazil. Regarding the curricula in Brazil, the medical schools have five to 6 years in duration, and student will have supervised and restricted contact with patients during the internship in the fourth year and on, without prior involvement with patients [43]. It was offered to the participants the possibility of a paper-and-pencil questionnaire or a QR code link for online participation. Those who adhered to the paper-and-pencil represented 18.3% of the sample, with a participation rate of 46.22% of the contacted clinicians and students. Data were transferred from paper to password-protected electronic devices only accessible to the authors. The online data collection was performed via electronic platform (SurveyMonkey®), which do not allow for multiple responses, was anonymous, and no personal information was obtained. Participants were also asked to share the link of the questionnaire to their personal contacts. All participants were volunteers, and no compensation was provided, according to Brazilian laws. Only questionnaires that were fully completed were considered for this study, exempting the need of handling missing data. Inclusion criteria were being a medical student or a physician and 18 years of age or older.

### Items generation

The questionnaire was developed and applied in Brazilian Portuguese. The items of the self-report Braking Bad News Attitude Scale (BBNAS) were generated a priori to evaluate two attitudes among clinicians: their level of agreement with the strategy recommended in the SPIKES protocol [16] to properly communicating bad news, and their agreement with the possibility to be trained to learn the desired skills. Accordingly, there are several attitudes in terms of a favorable or unfavorable relationship with an object, which can indicate positive or negative measures [44]. From this, we proposed an agreement/disagreement scale in view of the possibility of behaviors encouraged by SPIKES or training. Thus, the proposed instrument does not measure whether the individual actually performs a certain behavior (eg, “I encourage the patient to express their feelings and clarify their doubts”), but rather their relationship with that possible behavior (eg, how much the individual agrees to behave by encouraging the patient to express their feelings and clarify their doubts). The same occurs in the second domain of the “BBN training” domain (eg, degree of agreement, favorable position or not in relation to communication behaviors and the possibility of training them).

An initial pool of 15 items was generated by researchers and clinicians referring to the two dimensions described above with the main content taken from the SPIKES protocol. The SPIKES goal is to enable the clinician to fulfill the four most important objectives of the interview for disclosing bad news. These objectives include gathering information from the patient, providing medical information to the patient, offering him or her support, and eliciting the patient’s collaboration in developing a treatment plan. The name SPIKES is the acronym describing the six consecutive steps of a BBN conversation. Setting up (S) describes the process of preparing for the talk; Perception (P) is the parts in which the physician determines the patient’s perception of the situation; Invitation (I) refers to the invitation of the patient to receive the news; Knowledge (K) is the information breakout; Emotions (E) refer to the proper way of responding to patient’s emotions. Finally, Strategy and Summary (S) aim to ascertain whether the patient has adequately understood the situation and the treatment plan [16].

The 15 generated items were piloted among 30 residents. They were asked to evaluate the clarity of instructions and items, relevance, and appropriateness of items content in relation to the SPIKES protocol. The participants’ feedback was mostly related to the instructions and phrasing, and few suggestions regarding the conceptual correctness of the content were given in person. This final version was used in the present validation study.

### Measures

Participants responded to a survey containing a sociodemographic part including age, sex, and pediatrician or medical student position, the Breaking Bad News Attitude Scale (BBNAS), the Jefferson Scale of Physician Empathy (JSPE), and three questions to self-assess the participants’ BBN skills.

The BBNAS is the newly developed 15-item scale. Respondents are asked to rate how much they agreed with each statement using a 5-point scale from 0 (*strongly disagree*) to 4 (*strongly agree*). The BBNAS items are reported in detail (Additional file 1). A BBNAS score calculator is available as a spreadsheet (Additional file 2).

The JSPE [45] is a measure of the orientation of medical students and professionals toward empathy in patient-care situations. Items (e.g., “A physician who can view things from another person’s perspective can render better care”) are answered on a 7-point scale ranging from 1 (*strongly disagree*) to 7 (*strongly agree*). The Brazilian version [46] has 20 items referring to compassionate care (11 items), perspective taking, (2 items), and standing in the patient’s shoe (7 items). In this study a total summative score was calculated with Cronbach’s alpha of 0.76.

Three additional ad-hoc questions were used to assess how participants self-rated their BBN skills using a scale between 1 (*poor*) to 10 (*excellent*). Questions refer to three aspects of BBN: skills in conveying bad news, skills in dealing with emotions of the patient who received bad news, and ability to comfort the patient when informed about bad news.

### Data analysis

The 15 items of the BBNAS were preliminary evaluated for adequacy of skewness and kurtosis of residuals (between − 1 and + 1), and for discrimination power (D > 0.30).

A subsample of 200 participants was randomly extracted from the whole sample to run EFA, while the remaining 363 participants were used to run CFA. Random extraction of samples was generated by the SPSS command.

For the EFA, the factorability of the data was preliminary evaluated using the Kaiser-Meyer-Olkin test (KMO > 0.50), the Bartlett test of sphericity (*p* < 0.05), and the value of the matrix determinant (> 0.00001). The EFA extraction method was principal axis factoring, which is robust to violation of multivariate normality, with a direct Oblimin rotation with Kaiser normalization to allow for correlations between factors. The number of factors to be retained was determined by comparison between the initial and random eigenvalues calculated by parallel analysis (PA) with 500 replications. Criteria for inclusion of each item were primary factor loading >|0.30|, and meaningful and useful contribution to the target factor [47, 48].

The CFA intended to confirm the structure identified by the EFA with the maximum likelihood robust (MLR) estimator. The model fit was evaluated by chi-square (χ^2^ *p* < 0.05), CMIN discrepancy value < 3, Root Mean Square Error of Approximation (RMSEA) with 90% confidence interval (CI) between 0.05 and 0.08, Standardized Root Mean Square Residual (SRMR) < 0.08, and Comparative Fit Index (CFI) > 0.90. Akaike (AIC) and Sample-size adjusted Bayesian (SSABIC) comparative indices were also used to compare two different models estimates with lower values indicating a better fit. The difference of competitive models was calculated with the exact Satorra-Bentler difference test statistic (S-B χ^2^, *p* < 0.05) [49, 50].

Reliability was assessed as internal consistency with Cronbach’s alpha (≥0.70) and the composite reliability coefficient (CR) (≥0.80) [51].

Pearson’s correlation coefficient was used to correlate the BBNAS scores with age, the JSPE score, and the three questions used to self-assess BBN abilities. Analysis of variance (ANOVA) was used for differences across sexes and between pediatricians and medical students in the BBNAS scores [51]. The score obtained for each factor of the BBNAS was also transformed into percentage of agreement with the item statements, and results were interpreted based on tertiles.

Significance level was set at *p* < 0.05. Statistical analyses were performed with Mplus 8.3.

## Results

### Participant characteristics

A total of 563 participants completed the survey. Women were 78.5% (*n* = 442) and men were 21.5% (*n* = 121); pediatricians were 86% (*n* = 484) and medical students were 14% (*n* = 79). The mean age was 40.68 (SD = 13.36), ranging from 18 to 81 years. Most participants (58.1%, *n* = 327) were from the north or north-east regions of Brazil, 35.7% (*n* = 201) were from the south or southeast regions, while only 6.2% (*n* = 35) were from the western regions.

### Preliminary item analysis

In the total sample (*N* = 563) the values of skewness (− 0.49 to 0.13) and kurtosis (− 0.33 to 0.58) of residuals for each item varied within the appropriate range. Discrimination power of the items was between 0.48 and 0.70, and thus, all items were retained for the subsequent factor analyses. Values of skewness, kurtosis, and discrimination power for each item are reported in Supplementary Table 1 (Additional file 3)

### Exploratory factor analysis

Preliminary analyses on the first subsample of 200 participants indicated that the BBNAS data were suitable for EFA, showing a KMO value of 0.77, a Bartlett’s test value (χ^2^ = 675.10, DF = 105, *p* < 0.001), and a matrix determinant of 0.030, considered statistically significant.

Two factors corresponding to actual eigenvalues that were greater than the PA random eigenvalues were retained. See supplementary Table 2 (Additional file 4). From extraction of two factors a clear factor structure emerged (Table 1) explaining a total variance of 41.64%, with significant intercorrelation between factors (*r* = 0.52, *p* < 0.001). The first factor, explaining 34.08% of variance, included items 1, 2, 3, 4, 5, 6, 7, 8, 9, 10, and 13 and referred to the hypothesized dimension of attitude towards SPIKES strategy for BBN. The second factor, explaining 7.56% of variance, included items 11, 12, 14, and 15 and referred to the dimension of attitude towards BBN training. Reliability was considered good, with Cronbach’s alpha values of 0.85 (90% CI 0.82–0.88) for Factor 1 and 0.78 (90% CI 0.73–0.83) for Factor 2.

**Table 1:** Exploratory factor analysis of the BBNAS, items factor loadings

Item	Factor	
	1	2
1. Setting-up the place for bad news communication	**0.43**	0.23
2. Answering the patient without inhibiting him/her	**0.72**	
3. Assessing the patient’s perceptions about prognosis	**0.80**	
4. Establishing a trustworthy relationship with the patient	**0.53**	
5. Patients’ desire to discuss their case	**0.38**	
6. Planning a strategy to communicate bad news	**0.46**	
7. Answering the patient’s questions expressing support and respect	**0.77**	
8. Goal proposal and follow-up	**0.63**	
9. Encouraging the patient to express feelings and doubts	**0.75**	
10. Informing the family about psychological support	**0.42**	0.20
11. Consider necessity of improvements on skills	0.23	**0.49**
12. Advisable to receive training		**0.81**
13. Empathy assists in communication	**0.33**	0.25
14. Personal interest in courses and training		**0.71**
15. Communication can be trained		**0.66**
% of Variance explained	34.07	7.56
Cronbach’s alpha reliability	0.85	0.78

*BBNAS* Breaking Bad News Attitudes Scale. Exploratory factor analysis extraction method: principal axis factoring with direct Oblimin rotation and Kaiser normalization. Factor loadings < 0.20 were ommited

### Confirmatory factor analysis

**Fig. 1:**
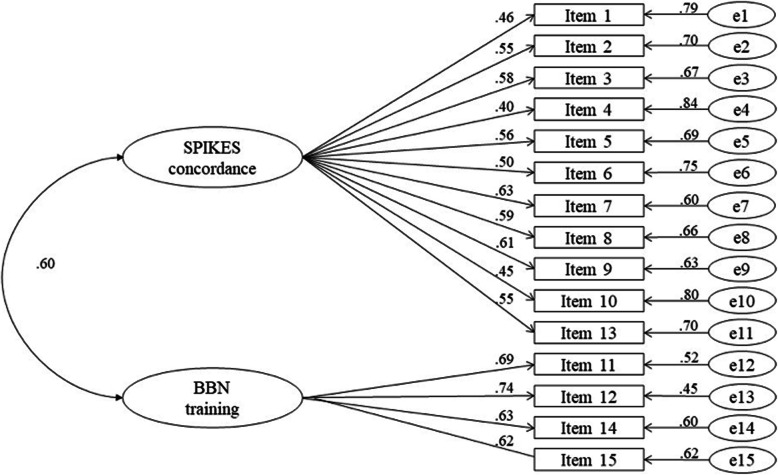
Measurement model of the BBNAS with standardized parameters. Legend BBNAS – Breaking Bad News Attitudes Scale; BBN – breaking bad news

Two models were tested with CFA, both based on the two factors emerged from EFA. The first one was a two-uncorrelated-factors model, while the second one was a two-correlated-factors model. The first model showed the following indices: χ^2^ = 240.31, DF = 90, *p* < 0.001; CMIN = 2.67; RMSEA = 0.07 (90% CI 0.06–0.08), SRMR = 0.14; CFI = 0.84, AIC = 11,312.33, and SSABIC = 11,344.82. The second model showed the following indices: χ^2^ = 163.82, DF = 89, *p* < 0.001; CMIN = 1.84; RMSEA = 0.05 (90% CI 0.04–0.06), SRMR = 0.05; CFI = 0.92, AIC = 11,220.722, and SSABIC = 11,253.927. S-B χ^2^ difference of the two models was 105.81, DF = 1, *p* < 0.001. The second model was preferred since it showed a better fit than the first model (Fig. 1). All parameters are considered statistically significant for the analysis proposed.

CFA confirmed a two-correlated-factor model for BBNAS, with Factor 1 named “SPIKES concordance” and Factor 2 named “BBN training”. Cronbach’s alpha was 0.81 (90% CI 0.77–0.83) for Factor 1, and 0.77 (90% CI 0.71–0.79) for Factor 2. CR was 0.87 for Factor 1, and 0.84 for Factor 2, considered good for this parameter.

### Descriptive statistics and associations with other variables

In the total sample (*N* = 563), the BBNAS score range was 0–44 for SPIKES concordance, with a mean value of 35.01 (SD = 5.46), and 0–16 for BBN training, with a mean value of 13.62 (SD = 2.43). Means and standard deviations of the 15 items are shown in Supplementary Table 1 (Additional file 3).

For each BBNAS factor the score obtained was also transformed into percentage of agreement with the SPIKES strategy or the BBN training, respectively, and two cut-points were suggested based on tertiles. Percentages of 0–33% were interpreted as *disagreement*, of 34–66% as *partial agreement*, and over 66% as *agreement*. Correspondence between percentages and raw scores is shown in Supplementary Table 3 (Additional file 5).

The SPIKES concordance factor correlated significantly and positively with the JSPE total score and with the three self-rated BBN skills items. The BBN training factor correlated significantly and positively with the JSPE total score, significantly and negative with the first self-rated BBN skills item, and non-significantly with the other two BBN skills items (Table 2).

**Table 2:** Pearson’s correlations of the BBNAS scores with other measures

	SPIKES concordance	BBN training
Jefferson Perspective Taking (total score)	0.24**	0.11*
SPS 1. Breaking bad news	0.15**	−0.11*
SPS 2. Dealing with the patient’s emotions	0.18**	−0.08
SPS 3. Comforting the patient	0.10*	−0.08

*BBNAS* Breaking Bad News Attitudes Scale. *SPS* Self-perceived skills related to deliverying bad news to patients

^*^p ≤ 0.05, ^**^p ≤ 0.001

Age was not associated with the SPIKES concordance factor (*r* = 0.03, *p* = 0.50), while it was significantly and negatively associated with BBN training factor (*r* = − 0.19, *p* ≤ 0.001).

Association with sex showed that women scored significantly higher than men in the SPIKES concordance factor. Association with the physician/student position showed that students scored significantly higher than physicians in the BBN training factor (Table 3).

**Table 3:** Associations of BBNAS scores with sex and physician/student position

	SPIKES concordance	BBN training
Women (*n* = 442)	35.39 (5.04)	13.73 (2.31)
Men (*n* = 121)	33.63 (7.03)	13.25 (2.80)
ANOVA results	F_(1,559)_ = 7.49; *p* = 0.006	F_(1,559)_ = 2.54, *p* = 0.11
Physician (*n* = 484)	34.93 (5.54)	13.49 (2.43)
Student (*n* = 79)	35.51 (4.93)	14.49 (1.97)
ANOVA results	F_(1,559)_ = 0.52, *p* = 0.47	F_(1,559)_ = 9.58, *p* = 0.002

*BBNAS* Breaking Bad News Attitudes Scale, *BBN* Breaking Bad News. Values are means and (standard deviation)

## Discussion

This study presents an instrument for assessing physicians’ agreement with the principles of the SPIKES protocol and with the possibility of being trained on BBN. Although patient’s preferences for BBN according to the SPIKES protocol has been recently measured [52], this is the first validation study of a measure of physicians’ attitudes towards the principles of the protocol.

The SPIKES protocol has been mainly discussed within the approach to oncology patients [16]. However, its principles and strategies for delivering bad news have been appropriately applied to other diseases such as the Down syndrome [53], child’s cleft lip [54], or cystic fibrosis [55]. We involved in this validation study pediatricians besides medical students, since there is evidence that bad news is frequently delivered inadequately, according to parents of pediatric patients [56].

It has been pointed out that the ability to communicate bad news may be affected by the setting as well as by the professionals’ psychological conditions and conceptions of what the procedure should be like [57]. We addressed the last issue by assessing how the participants considered BBN and the possibility to learn BBN skills.

The instrument developed in this study, named the Breaking Bad News Attitude Scale (BBNAS), showed good psychometric properties and appeared to be adequate to measure the attitudes of pediatricians and medical students regarding BBN. It is important to clarify that the BBNAS is not a tool for assessing knowledge of the SPIKES protocol, but it rather evaluates how much the physician/student agrees with the SPIKES values and principles, which have been recognized as essential for BBN. Clinicians might indeed have relevant knowledge about the protocol, but this does not necessarily reflect in their positive attitudes toward its implementation. On the other hand, the expectation was that a professional/student who agrees with the protocol could be potentially more receptive to adopt it. Indeed, the proposed instrument was able to measure the attitude towards training behaviors of giving bad news, considering the relationship that the individual has with the need to improve communication and receive training. The high score in this domain indicates that the participants positively consider the possibility of participating in training for this skill. Such availability is important, as it can identify a guiding line for training, as guide the design of training interventions promoting the ability to learn. Thus, training can be the way to provide better results in the development of skills [58, 59], specifically those related to communicating bad news.

It was expected that the score obtained in the SPIKES concordance factor indicated how much the practitioner agrees with the protocol, regardless of considering the desired skills as learnable. The non-overlap between the two BBNAS factors, although significantly intercorrelated, seems to confirm this expectation. Agreement with the SPIKES principles was also expected to be linked but distinguishable from self-perceived skills to delivery bad news. Indeed, the associations found in this study between these dimensions were significant but of small magnitude. Conversely, a small negative association was found between self-perceived BBN skills and a positive attitude towards BBN training, as we could reasonably expect. This seems to converge with data that showed that more experienced trainees are more likely to avoid bad news communication with patients [7].

Another expectation was that a positive attitude towards the SPIKES strategy would be associated with individual psychological characteristics such as empathy [20, 23], which has been also considered as an aspect of patient centered care [60]. Significant positive correlations of the BBNAS with the JSPE confirmed this aspect. Regarding the larger agreement of females with the principles of the SPIKES protocol, compared to males, it could be related to the general females’ higher empathy in the clinical setting [40], but this difference should be investigated more in depth and confirmed in future studies.

The agreement with the possibility to be trained in BBN is a critical point that has been measured by the BBNAS. As we could expect, medical students were more likely to consider BBN training than physicians. This result is consistent with a recent study [25] where doctors with more of 10 or 20 years of experience since graduation perceived themselves as better qualified to provide bad news than their younger colleagues. It is also reported that previous personal experiences may affect the attitudes regarding physician-patient communication [61].

The need for educational interventions, training, and identification of skills required for BBN has been frequently pointed out [e.g., [25]]. Several strategies have been proposed, and difficulties in evaluating these abilities have been acknowledged. Indeed, a recent meta-analysis showed that a variety of effective training in BBN had been introduced, but the heterogeneity of the results presented was high [23]. In addition, evaluation results examining the use of the protocols varied [62], and patient preferences on how to receive bad news seem to interfere in the BBN process [18]. Professionals who perceive themselves as capable of communicating bad news can do so effectively by depending upon their intuitive talents [63]. Unfortunately, if this self-perception is imprecise, their inadequate BBN behavior could have consequences for the patient. In this regard, detecting the levels of agreement by clinicians with BBN training is relevant to the development, planning, and success of effective educational interventions. In fact, individuals who consider certain skills as learnable and trainable may be available to participate in continuing education programs and be receptive to the proposed strategies.

The present study has several limitations. First, the self-assessment nature of the BBNAS could be criticized, because of the attendant possibility of reporting biases. However, other strategies, based on various sources of observable information, like, for example, the Implicit Association Test (IAT), are not very practical to work with, especially when involving large samples. Second, the small number of medical students involved in the study and the overrepresentation of females, that did not allow to assess invariance of the measurement across gender and physician/student position. Third, the sample was not representative of the country population, since there was a prevalence of participants from the north or northeast regions of Brazil. Fourth, test-retest reliability of the BBNAS was not assessed. Finally, the limited response rate is expected since there is no compensation for volunteers for participation by any means. Therefore, the BBNAS psychometric characteristics are to be further investigated using a larger and more balanced sample across physicians/students, sexes, and geographical areas, and other specialty physicians should be involved. Furthermore, longitudinal studies are needed to assess the BBNAS sensibility to change and the related usefulness in the context of continuing education.

## Conclusion

The BBNAS is a newly developed scale to measure the level of agreement that physicians and medical students have with the principles of the SPIKES protocol to deliver bad news. It allows measuring attitudes towards BBN as described by the SPIKES protocol and towards training to learn and implement BBN strategies. Its psychometric characteristics are promising and encourage the use of this measure in other medical contexts.

The information that can be collected by the BBNAS is relevant to the development and planning of effective educational interventions in BBN both for medical students and professionals. Indeed, individuals who have a positive attitude towards the strategy to better communicate bad news to patients and are favorable to training for the delivery of bad news could be more supportive of the strategies proposed in the training process. Efficiency in BBN may benefit both patient and parents and has the potential to change the way how the news is received. It may also enhance patients’ adherence to treatment, their physical and mental health outcomes and decreases physicians’ levels of stress generated by the necessity to communicate bad news. These gains can be promoted through continuing education ensuring that the educational interventions are planned, executed, and evaluated based on evidence.

## Supplementary Information

**Additional file 1.** The Breaking Bad New Attitude Scale (BBNAS) content of items.

**Additional file 2.** BBNAS Scoring Calculator. A spreadsheet to calculate the BBNAS score.

**Additional file 3: Supplementary Table 1.** Descriptive statistics of items of the Breaking Bad News Attitude Scale.

**Additional file 4: Supplementary Table 2.** Parallel analysis for exploratory factor analysis of the BBNAS.

**Additional file 5: Supplementary Table 3.** Correspondence between percentage and raw scores of the Breaking Bad News Attitudes Scale.

## Data Availability

The datasets used and/or analyzed during the current study are available from the corresponding author on reasonable request.

## Abbreviations

AIC: Akaike comparative index
ANOVA: Analysis of variance
BBN: Breaking bad news
BBNAS: Breaking bad news assessment scale
CFA: Confirmatory factor analysis
CFI: Comparative fit index
CR: Composite reliability coefficient
EFA: Exploratory factor analysis
JSPE: Jefferson Scale of Physician Empathy
MLR: Maximum likelihood robust estimator
RMSEA: Root mean square error of approximation
S-B χ^2^: Exact Satorra-Bentler difference test
SRMR: Standardized root mean square residual
SSABIC: Sample-size adjusted Bayesian comparative index

## References

[1] Buckman R (1992). Breaking bad news: a guide for health care professionals.

[2] Bousquet G, Orri M, Winterman S, Brugière C, Verneuil L, Revah-Levy A (2015). Breaking bad news in oncology: a meta-synthesis. J Clin Oncol.

[3] Hollyday SL, Buonocore D (2015). Breaking bad news and discussing goals of care in the intensive care unit: AACN. Adv Crit Care.

[4] Fallowfield L, Jenkins V (2004). Communicating sad, bad, and difficult news in medicine. Lancet..

[5] Maynard DW (1996). On "realization" in everyday life: the forecasting of bad news as a social relation. Am Sociol Rev.

[6] Studer RK, Danuser B, Gomez P (2017). Physicians’ psychophysiological stress reaction in medical communication of bad news: a critical literature review. Int J Psychophysiol.

[7] Sikstrom L, Saikaly R, Ferguson G, Mosher PJ, Bonato S, Soklaridis S (2019). Being there: a scoping review of grief support training in medical education. Fernández-Alcántara M, editor. PLoS One.

[8] Wolfe AD, Frierdich SA, Wish J, Kilgore-Carlin J, Plotkin JA, Hoover-Regan M (2014). Sharing life-altering information: development of pediatric hospital guidelines and team training. J Palliat Med.

[9] Finan C, Nasr SZ, Rothwell E, Tarini BA (2015). Primary care providers’ experiences notifying parents of cystic fibrosis newborn screening results. Clin Pediatr (Phila).

[10] Rider EA, Volkan K, Hafler JP (2008). Pediatric residents’ perceptions of communication competencies: implications for teaching. Med Teach..

[11] Kelley KJ, Kelley MF (2013). Teaching empathy and other compassion-based communication skills. J Nurs Prof Dev.

[12] Girgis A, Sanson-Fisher RW (1998). Breaking bad news 1: current best advice for clinicians. Behav Med.

[13] Villagran M, Goldsmith J, Wittenberg-Lyles E, Baldwin P, Creating COMFORT (2010). A communication-based model for breaking bad news. Commun Educ.

[14] Narayanan V, Bista B, Koshy C (2010). ‘BREAKS’ protocol for breaking bad news. Indian J Palliat Care.

[15] Keefe-Cooperman K, Brady-Amoon P (2013). Breaking bad news in counseling: applying the PEWTER model in the school setting. J Creativ Ment Health.

[16] Baile WF, Buckman R, Lenzi R, Glober G, Beale EA, Kudelka AP (2000). SPIKES-A six-step protocol for delivering bad news: application to the patient with Cancer. Oncologist.

[17] Buckman R (2001). Communication skills in palliative care. Neurol Clin.

[18] Mirza RD, Ren M, Agarwal A, Guyatt GH (2018). Assessing patient perspectives on receiving bad news: a survey of 1337 patients with life-changing diagnoses. AJOB Empir Bioeth.

[19] Leone D, Menichetti J, Barusi L, Chelo E, Costa M, De Lauretis L, et al. Breaking bad news in assisted reproductive technology: a proposal for guidelines. Reprod Health. 2017;14(1):1–10. 10.1186/s12978-017-0350-1. 10.1186/s12978-017-0350-1 PMC5520370 28728610

[20] Marschollek P, Bąkowska K, Bąkowski W, Marschollek K, Tarkowski R (2019). Oncologists and breaking bad news—from the informed patients’ point of view. The evaluation of the SPIKES protocol implementation. J Cancer Educ.

[21] Schmid Mast M, Kindlimann A, Langewitz W (2005). Recipients’ perspective on breaking bad news: how you put it really makes a difference. Patient Educ Couns.

[22] Gorniewicz J, Floyd M, Krishnan K, Bishop TW, Tudiver F, Lang F (2017). Breaking bad news to patients with cancer: a randomized control trial of a brief communication skills training module incorporating the stories and preferences of actual patients. Patient Educ Couns.

[23] Johnson J, Panagioti M (2018). Interventions to improve the breaking of bad or difficult news by physicians, medical students, and interns/residents: a systematic review and meta-analysis. Acad Med.

[24] Vermylen JH, Wood GJ, Cohen ER, Barsuk JH, McGaghie WC, Wayne DB (2019). Development of a simulation-based mastery learning curriculum for breaking bad news. J Pain Symptom Manag.

[25] Ferreira da Silveira FJ, Botelho CC, Valadão CC (2017). Breaking bad news: doctors’ skills in communicating with patients. Sao Paulo Med J.

[26] Ahmed SA, Ashry SK, Widdershoven G (2019). Effectiveness of online teaching for development of resident beliefs and understandings: a study on breaking bad news to patients. Health Profess Educ.

[27] Rushton J, Hicks PJ, Carraccio CL (2010). The next phase of pediatric residency education: the partnership of the milestones project. Acad Pediatr.

[28] Swing SR (2007). The ACGME outcome project: retrospective and prospective. Med Teach..

[29] Komatz K, Zayas J (2012). Using a simulation lab to deliver pediatric bad news. J Pain Symptom Manag.

[30] Turner DA, Mink RB, Lee KJ, Winkler MK, Ross SL, Hornik CP, et al. Are pediatric critical care medicine fellowships teaching and evaluating communication and professionalism? Pediatr Crit Care Med. 2013;14(5):454–61. 10.1097/PCC.0b013e31828a746c. 10.1097/PCC.0b013e31828a746c PMC4112058 23867427

[31] Milton AC, Mullan B (2017). Views and experience of communication when receiving a serious mental health diagnosis: satisfaction levels, communication preferences, and acceptability of the SPIKES protocol. J Mental Health.

[32] Reed S, Kassis K, Nagel R, Verbeck N, Mahan JD, Shell R (2015). Breaking bad news is a teachable skill in pediatric residents: a feasibility study of an educational intervention. Patient Educ Couns.

[33] Dias LM, Carvalho AEV, Furlaneto IP, de Oliveira CGS (2018). Medical residents perceptions of communication skills, a workshop on breaking bad news. Rev Bras Educ Medica.

[34] Lamba S, Tyrie LS, Bryczkowski S, Nagurka R (2016). Teaching surgery residents the skills to communicate difficult news to patient and family members: a literature review. J Palliat Med.

[35] Clayton JM, Butow PN, Waters A, Laidsaar-Powell RC, O’Brien A, Boyle F, et al. Evaluation of a novel individualised communication-skills training intervention to improve doctors’ confidence and skills in end-of-life communication. Palliat Med. 2013;27(3):236–43. Available from:. 10.1177/0269216312449683. 10.1177/0269216312449683 22711714

[36] Huntley CD, Salmon P, Fisher PL, Fletcher I, Young B (2012). LUCAS: a theoretically informed instrument to assess clinical communication in objective structured clinical examinations: LUCAS: an instrument to assess communication. Med Educ.

[37] Schildmann J, Kupfer S, Burchardi N, Vollmann J (2012). Teaching and evaluating breaking bad news: a pre–post evaluation study of a teaching intervention for medical students and a comparative analysis of different measurement instruments and raters. Patient Educ Couns.

[38] van Weel-Baumgarten EM, Brouwers M, Grosfeld F, Hermus FJ, Van Dalen J, Bonke B (2012). Teaching and training in breaking bad news at the dutch medical schools: a comparison. Med Teach.

[39] Rosenberg M (1960). Attitude organization and change: an analysis of consistency among attitude components.

[40] Hojat M, Gonnella JS, Mangione S, Nasca TJ, Veloski JJ, Erdmann JB, et al. Empathy in medical students as related to academic performance, clinical competence and gender: empathy in medical students. Med Educ. 2002;36(6):522–7. Available from:. 10.1046/j.1365-2923.2002.01234.x. 10.1046/j.1365-2923.2002.01234.x 12047665

[41] Borasino S, Morrison W, Silberman J, Nelson RM, Feudtner C. Physicians’ contact with families after the death of pediatric patients: a survey of pediatric critical care practioners’ beliefs and self-reported practices. Pediatrics. 2008;122(6):e1174–8. 10.1542/peds.2008-0952. 10.1542/peds.2008-0952 19015203

[42] Wolf EJ, Harrington KM, Clark SL, Miller MW (2013). Sample size requirements for structural equation models: an evaluation of power, bias, and solution propriety. Educ Psychol Meas.

[43] dos Santos RA, Tenorio Nunes MDP (2019). Medical education in Brazil. Med Teach.

[44] Thurstone LL (1931). The measurement of attitudes. J Abnorm Soc Psychol.

[45] Hojat M, Mangione S, Nasca TJ, Cohen MJM, Gonnella JS, Erdmann JB, et al. The Jefferson scale of physician empathy: development and preliminary psychometric data. Educ Psychol Meas. 2001;61(2):349–65. 10.1177/00131640121971158.

[46] Paro HB, Daud-Gallotti RM, Tibério IC, Pinto RMC, Martins MA (2012). Brazilian version of the Jefferson scale of empathy: psychometric properties and factor analysis. BMC Med Educ.

[47] Williams B, Onsman A, Brown T (2010). Exploratory factor analysis: a five-step guide for novices. Australas J Paramedicine.

[48] Henson RK, Roberts JK (2006). (). Use of exploratory factor analysis in published research: common errors and some comment on improved practice. Educ Psychol Meas.

[49] Hu L, Bentler PM (1999). Cutoff criteria for fit indexes in covariance structure analysis: conventional criteria versus new alternatives. Struct Equ Model Multidiscip J.

[50] Tabachnick BG, Fidell LS. Using Multivariates Statistics. 5th ed. Boston: Pearson Education, Inc; 2007.

[51] Cohen LH, Towbes LC, Flocco R (1988). Effects of induced mood on self-reported life events and perceived and received social support. J Pers Soc Psychol.

[52] von Blanckenburg P, Hofmann M, Rief W, Seifart U, Seifart C. Assessing patients’ preferences for breaking bad news according to the spikes-protocol: the mabban scale. Patient Educ Couns. 2020:S0738399120301105. 10.1016/j.pec.2020.02.036. 10.1016/j.pec.2020.02.036 32151521

[53] Skotko BG, Capone GT, Kishnani PS (2019). Down syndrome diagnosis study group, postnatal diagnosis of down syndrome: synthesis of the evidence on how best to deliver the news. Pediatrics..

[54] Strauss RP, Sharp MC, Lorch SC, Kachalia B (1995). Physicians and the communication of ‘bad news’: parent experiences of being informed of their child’s cleft lip and/or palate. Pediatrics..

[55] Havermans T, Tack J, Vertommen A, Proesmans A, de Boeck K (2015). Breaking bad news, the diagnosis of cystic fibrosis in childhood. J Cyst Fibros.

[56] Contro N, Larson J, Scofield S, Sourkes B, Cohen H (2002). Family perspectives on the quality of pediatric palliative care. Archiv Pediat Adol Med.

[57] Rosenbaum ME, Ferguson KG, Lobas JG (2004). Teaching medical students and residents skills for delivering bad news: a review of strategies. Acad Med.

[58] Engeström Y (1994). Training for change: new approach to instruction and learning in working life.

[59] Hwang A (2003). Training strategies in the management of knowledge. J Knowl Manage.

[60] Gremigni P, Casu G, Sommaruga M (2016). Dealing with patients in healthcare: a self-assessment tool. Patient Educ Couns.

[61] Farber NJ, Urban SY, Collier VU, Weiner J, Polite RG, Davis EB, et al. The good news about giving bad news to patients. J Gen Intern Med. 2002;17(12):914–22. 10.1046/j.1525-1497.2002.20420.x. 10.1046/j.1525-1497.2002.20420.x PMC1495144 12472927

[62] Seifart C, Hofmann M, Bär T, Riera Knorrenschild J, Seifart U, Rief W (2014). Breaking bad news–what patients want and what they get: evaluating the SPIKES protocol in Germany. Ann Oncol.

[63] Carroll C, Carroll C, Goloff N, Pitt MB (2018). When bad news isn’t necessarily bad: recognizing provider bias when sharing unexpected news. Am Acad Pediatrics.

