# Supplementary material for: Development and validation of The Breaking Bad News Attitudes Scale
Source: BMC Med Educ. 2021 Apr 7;21:196. doi: 10.1186/s12909-021-02636-5 (PMC8028222; doi:10.1186/s12909-021-02636-5)
Supplement: Supplementary file 1 — Additional file 1. The Breaking Bad New Attitude Scale (BBNAS) content of items. [file 12909_2021_2636_MOESM1_ESM.pdf]

Additional file 1

The Breaking Bead New Attitude Scale (BBNAS) content of items

Factor 1 – SPIKES concordance

1. I prepare a suitable place when I need to transmit bad news.
2. After reporting bad news, I try to answer the questions without inhibiting this moment of unpredictable reactions.
3. I try to understand if the patient was informed about his or her prognosis.
4. I only report bad news after establishing a relationship of trust with the patient.
5. It is important to know if the patient wants to discuss his pathology and consequences.
6. I organize some strategy in advance to convey bad news.
7. After giving bad news, I answer the patient's questions showing support, respect and understanding.
8. I end the communication of the bad news proposing a plan of future goals for follow-up.
9. After communicating the bad news, I encourage the patient to express their feelings and clarify their doubts.
10. I always inform the family that there will be psychological support when necessary.

13. Medical empathy can help in the transmission of bad news.

Factor 2 – Training

11. I believe there should be improvements and/or more investment in improving communication skills of bad news.
12. It would be desirable to receive training to report bad news.

14. I am interested in courses and training on bad news information.
15. The transmission of bad news is a skill that can be trained and stimulated.

Response is based on a 5-point scale from 0 = *strongly disagree* to 4 = *strongly agree.*
