# Supplementary material for: Development and validation of The Breaking Bad News Attitudes Scale
Source: BMC Med Educ. 2021 Apr 7;21:196. doi: 10.1186/s12909-021-02636-5 (PMC8028222; doi:10.1186/s12909-021-02636-5)
Supplement: Supplementary file 3 — Additional file 3: Supplementary Table 1. Descriptive statistics of items of the Breaking Bad News Attitude Scale. [file 12909_2021_2636_MOESM3_ESM.pdf]

Additional file 3

Supplementary Table 1

Descriptive statistics of items of the Breaking Bad News Attitude Scale (N = 563)

Item	Skewness of residual	Kurtosis of residual	D-value	Mean (Standard Deviation)
Item 1	-0.487	0.577	0.60	3.22 (0.84)
Item 2	-0.482	0.419	0.55	3.38 (0.71)
Item 3	-0.462	-0.214	0.54	3.38 (0.66)
Item 4	-0.280	-0.211	0.70	2.60 (1.01)
Item 5	0.013	0.073	0.58	3.35 (0.74)
Item 6	-0.293	0.379	0.64	2.78 (0.93)
Item 7	-0.144	0.398	0.48	3.59 (0.64)
Item 8	0.036	0.029	0.66	2.96 (0.94)
Item 9	-0.232	-0.005	0.59	3.31 (0.77)
Item 10	-0.386	-0.200	0.65	3.06 (0.90)
Item 11	-0.373	0.215	0.41	3.66 (0.65)
Item 12	-0.042	-0.200	0.50	3.54 (0.76)
Item 13	0.131	-0.194	0.58	3.38 (0.78)
Item 14	-0.270	-0.427	0.66	3.11 (0.94)
Item 15	-0.147	-0.333	0.58	3.31 (0.80)
