# Supplementary material for: Development and validation of The Breaking Bad News Attitudes Scale
Source: BMC Med Educ. 2021 Apr 7;21:196. doi: 10.1186/s12909-021-02636-5 (PMC8028222; doi:10.1186/s12909-021-02636-5)
Supplement: Supplementary file 4 — Additional file 4: Supplementary Table 2. Parallel analysis for exploratory factor analysis of the BBNAS. [file 12909_2021_2636_MOESM4_ESM.pdf]

Additional file 4

Supplementary Table 2

Parallel analysis for exploratory factor analysis of the BBNAS (n = 200)

Item	Actual Eigenvalues	Random Eigenvalues
Item 1	5.668	1.498
Item 2	1.633	1.379
Item 3	1.034	1.295
Item 4	0.900	1.221
Item 5	0.892	1.154
Item 6	0.710	1.092
Item 7	0.703	1.038
Item 8	0.607	0.981
Item 9	0.561	0.926
Item 10	0.522	0.874
Item 11	0.413	0.819
Item 12	0.403	0.767
Item 13	0.362	0.713
Item 14	0.337	0.656
Item 15	0.256	0.587

BBNAS = Breaking Bad News Attitude Scale. Number of replications was 500.
