# Supplementary material for: Development and validation of The Breaking Bad News Attitudes Scale
Source: BMC Med Educ. 2021 Apr 7;21:196. doi: 10.1186/s12909-021-02636-5 (PMC8028222; doi:10.1186/s12909-021-02636-5)
Supplement: Supplementary file 5 — Additional file 5: Supplementary Table 3. Correspondence between percentage and raw scores of the Breaking Bad News Attitudes Scale. [file 12909_2021_2636_MOESM5_ESM.pdf]

Additional file 5

Supplementary Table 3

Correspondence between percentage and raw scores of the Breaking Bad News Attitudes Scale

Each factor was scored summing up the pertaining items, thus, Factor 1 score was 0-44 and Factor 2 score was 0-16. Then the raw scores were transformed into percentage. The suggested cut-points for each factor based on tertiles (i.e., the two points that divide the distribution of scores into three parts, each containing a third of the population) were 14 and 28 for Factor 1, and 7 and 10 for Factor 2. A value below the first cut-point is representative of *disagreement* (0% to 33%); a value between the first and the second cut-points is representative of *partial agreement* (34% to 66%), and a value above the second cut-point represents *agreement* (over 66%).

SPIKES concordance		BBN training	
Raw score	Percent (%)	Raw score	Percent (%)
0	0	0	0
1	2,3	1	6,3
2	4,5	2	12,5
3	6,8	3	18,8
4	9,1	4	25,0
5	11,4	**5**	31,3
6	13,6	6	37,5
7	15,9	**7**	43,8
8	18,2	8	50,0
9	20,5	9	56,3
10	22,7	**10**	62,5
11	25,0	11	68,8
12	27,3	12	75,0
13	29,5	13	81,3
**14**	31,8	14	87,5

15	34,1
16	36,4
17	38,6
18	40,9
19	43,2
20	45,5
21	47,7
22	50,0
23	52,3
24	54,5
25	56,8
26	59,1
27	61,4
**28**	63,6
29	65,9
30	68,2
31	70,5
32	72,7
33	75,0
34	77,3
35	79,5
36	81,8
37	84,1
38	86,4
39	88,6
40	90,9
41	93,2
42	95,5
43	97,7
44	100,0

15	93,8
16	100,0
